# Epidemiology of childhood cancer

**DOI:** 10.1186/1476-069X-10-S1-S8

**Published:** 2011-04-05

**Authors:** Benedetto Terracini

**Affiliations:** 1 Center for Cancer Prevention, University of Torino, San Giovanni Battista University Hospital, Italy

## Abstract

At least in economically developed countries, in the last decades, the incidence of childhood cancer has increased and the increase is unlikely to be an artefact. Causes of the increase have not been identified: a role of preventable environmental exposures is possible. Changes have also occurred in the age distribution of acute lymphoblastic leukaemia.

Currently, children with cancer can be successfully treated and cured. However, access to the best therapy differs widely among countries because of the unequal distribution of resources for cancer care. Any double standard in the fate of children with cancer is ethically unacceptable.

## Introduction

Two main reasons drove Renzo Tomatis’ attention for the epidemiology of childhood cancer: the relevance of transgenerational carcinogenesis to cancer prevention and the unacceptability, for ethical reasons, of any double standard in the fate of children with cancer.

Tomatis’ concern was that an initiating event could be inherited by subsequent generations and revealed after postnatal exposure to mutagens/carcinogens or even non genotoxic agents. This has important ramifications for humans, since humans are exposed throughout life to many environmental factors that may in various ways enhance the progression of cancer [1]. The current focus on epigenetic mechanisms in carcinogenesesis, including pediatric cancer [2] points in the same direction. This concern of Renzo was also at the basis of his stress on “possible” carcinogenic agents (i.e. IARC category 2B). The fact that one or more steps of the carcinogenic process may occur as distant in time as in previous generations adds further difficulty to correctly assessing risk [3]. Further, Tomatis emphasized a paradox. The hypothesis that cancer is multifactorial in origin is generally agreed, but agencies assess risk for individual carcinogenic agents [4]. It is to be wondered whether epidemiological studies give sufficient attention to interaction between different environmental agents and between environmental agents, social pressure, behaviour (and, when applicable, hereditary factors).

## Time trends in the incidence of childhood cancer

Several studies in industrialized countries have estimated that the incidence of cancer before age 15 has increased during the last decades of the 20^th^ century and early in the current millennium. This has been shown, among others, by the Surveillance, Epidemiology and End Results (SEER) Programme s in the US [5] and by a large European multicentric study coordinated by the International Agency for Research on Cancer [6]. In Europe, rates have increased in the order of 1-2% per year, the increment regards most cancer types and has involved also adolescents (age 15-19).

The finding can hardly be attributed to improvements in diagnostic procedures, the quality of which - in the countries where the increase has been noticed - has been high and fairly constant since more than a quarter century. In most studies, and particularly in the large IARC study [6] the conventional indexes of quality of registration were reported to have been constant over the considered period. Indeed, given the rarity of childhood cancer (in the Western world, one child out of 500 develops a cancer before age 15), bias might derive from the loss of a few cases in the early year s of activity of cancer registries. However, the reproducibility of findings between estimates from independent studies on different populations indicates that the evidence for an increase is sufficiently strong as to cause concern. As yet, no reports on time trends from childhood cancer registries located in less reach countries are available.

If an artefact is to be excluded, the increase can hardly be attributed to anything else than environmental factors, in the broadest sense of the term. A role of infection and/or immunological changes has been postulated for the increase of acute lymphoblastic leukaemia [7,8], which is less plausible for solid cancers.

There is another epidemiological feature of childhood acute lymphoid leukaemia (ALL) which seems to affected by both genetic and environmental determinants: the peak of age specific incidence in age 2-3 compared to oldest and youngest children.- The peak was described on mortality data in the UK half a century ago [9]. In those days, leukaemia was highly lethal and mortality was a good proxy for incidence. In comparing subsequent cohorts of birth, Court Brown and Doll noticed that in the UK the peak started to appear in children born around 1935 and became progressively more obvious in the subsequent cohorts of birth. In the US, half a century ago, the peak was obvious in white but not in black children. Neither it was obvious from Japanese mortality statistics. Much more recently, comparison of data from European cancer registries over the three decades between 1970 and 2000 showed that in all periods the peak was less obvious in Eastern than in Western European countries, but also that the difference tended to decrease in time.

A peak in such circumscribed period of life suggests that there is a period (prenatal?) of particular susceptibility to a cellular mutation. The association of the peak with important socioeconomic changes suggests that in a few decades changes have occurred either in individual susceptibility or in exposure to exogenous agents. However, the possible mechanism underlying such changes remains to be found.

## The gap in the chances of being cured of a childhood cancer

Worldwide, every year, the number of children being diagnosed a cancer before reaching age 15 exceeds 200,000. Four fifths of them live in low income countries: given the low birth rate in economically developed countries, this proportion is expected to increase to 90% in a few years. Nowadays, in the economically developed countries , approximately 80% children with cancer survive (it was less than 20% half a century ago). The corresponding proportion is much lower in low income countries, where barriers are found in all steps of cancer care, ranging between availability of facilities for recognition of cancer cases and access to expensive therapeutic protocols [10]. Since almost two decades, the moral duty of ensuring the same rights to children with cancer all over the world has been a strong point of the International Society of Pediatric Oncology.

Tomatis was well aware of this double standard and that the high cost of cancer therapy impairs equity for all to be cured of a cancer. In his words “this is perhaps the most powerful argument in support of primary prevention of cancer “ [11].

## Some conclusions

Most of the available information on etiological risk factors for childhood cancer derives from case-control studies: this is to be expected, given the rarity of childhood cancer. As yet, drawing conclusions from such studies is impaired by several circumstances, such as methodological limitations of each study, heterogeneity of the criteria for assessing and estimating parental and children’s exposures (and assessment of the role that non differential misclassification might have in “negative” studies), heterogeneity in the criteria for selecting controls publication bias. There is no doubt that more research is needed in order to understand the mechanism behind the creasing trend of childhood cancer incidence reported by cancer registries in a consistent part of the world. On the other hand, many agents which have been hypothesized to have a role in the development of cancer in children (such as vehicular and industrial emissions, sedentariness, EMF, fast food etc) are also associated with other paediatric conditions. The evidence for causality for associations with non neoplastic conditions is as strong and perhaps stronger than the corresponding evidence of an association with cancer. It is to be wondered whether it is actually necessary to wait for a stronger evidence of causality for cancer in order to implement primary prevention measures.

Finally, Tomatis would have endorsed the “Erice statement” of pediatric oncologists stating that the long term goal of the cure and care of the child with cancer is that he/she becomes a resilient, fully functioning adult with an optimal health-related quality of life, accepted in the society at the same level of his/her age partners [12].

## Competing interests

The author declare that has no competing financial or non-financial interests
